# Defining Delayed Discharges of Inpatients and Their Impact in Acute Hospital Care: A Scoping Review

**DOI:** 10.34172/ijhpm.2020.94

**Published:** 2020-06-29

**Authors:** Alexander Micallef, Sandra C. Buttigieg, Gianpaolo Tomaselli, Lalit Garg

**Affiliations:** ^1^Department of Health Services Management, Faculty of Health Sciences, University of Malta, Msida, Malta.; ^2^Department of Computer Information Systems, Faculty of ICT, University of Malta, Msida, Malta.

**Keywords:** Acute Hospitals, Bed-Blocking, Delayed Discharges, Hospital Inpatients Flow, Transition of Care

## Abstract

**Background:** With the ever-increasing demand on acute healthcare, the hospital discharge process and delayed discharges are considered relevant in achieving optimal performance in clinical settings. The purpose of this paper is to review the literature to identify conceptual and operational definitions of delayed discharges, identify causes and effects of delayed discharges, and also to explore the literature for interventions aimed at decreasing the impact (in terms of reducing the number/rate of delays) of delayed discharges in acute healthcare settings.

**Methods:** An extensive literature search yielded a total of 26 248 records. Sixty-four research articles were included in the scoping review after considering inclusion/exclusion criteria and the PRISMA (Preferred Reporting Items for Systematic Reviews and Meta-Analysis) search strategy. The following databases were utilized: Cochrane, EBSCO, PubMed, PubMed Central, Medline, and Web of Science. The search was carried out between January 2017 and March 2020 and covered literature ranging from 1990 to 2019. Results were reviewed by authors for duplicates and filtered using the inclusion/ exclusion criteria. Tables were created to classify the chosen articles (n = 64), allowing us to organise findings and results.

**Results:** Conceptual and operational definitions were analysed. In turn, causes and effects of delayed discharges were extracted and represented in diagrammatic format, together with specific interventions used in acute healthcare settings to lessen the effect of delayed discharges. Operational definitions of delayed discharges were found to be more difficult to establish, particularly in the light of the vast number of different scenarios and workplace interventions uncovered in the literature. The main causes of delayed discharges were faulty organisational management, inadequate discharge planning, transfer of care problems, and age. The main effects were bed-blocking, A&E (Accident & Emergency) overcrowding, and financial implications. The main interventions included ‘discharge before noon’ initiative, ‘discharge facilitation tools,’ ‘discharge delay tracking’ mechanisms, and the role of general practitioners and social care staff.

**Conclusion:** This paper fills a gap in the fragmented literature on delayed inpatient discharges by providing a researchbased perspective on conceptual and operational definitions, causes and effects, as well as interventions to minimize their impact. The findings and definitions are intended as points of reference for future research.

## Introduction

Over the past few decades, healthcare systems in both developed and developing countries have been subjected to increasingly challenging financial scenarios, more so against the background of the 2008-2009 financial and macro-economic crises.^1^ Specifically, European Union (EU) countries are increasingly focusing their efforts on an overall reduction in public sector expenditure on healthcare through the elimination of resource waste and inefficiency.^2^ Such issues have mainly revolved around events related to the admission and discharge of patients in acute hospital settings. The efficiency of hospital processes, in relation to the admission and discharge processes, has attracted the attention of scholars in the field of health services in a bid to ensure efficiency and effectiveness without jeopardizing quality of care.^2^

The hospital discharge process stands at the core of such issues. Healthcare organisations are complex and unique, meaning that understanding the behaviour of each system is crucial in the attempt to manage it effectively.^3^ It is of utmost importance that hospital discharges are not viewed as some ‘end point’ but rather as another step in the patient pathway through acute hospital care.^4^ Various stakeholders are involved in the provision and co-ordination of healthcare in this transition stage so as to ensure safe transfer of care. Clinical pathways are highly intricate because they are often unique to individual patients going through the pathways. The delayed discharge of hospital patients has been singled out as a major factor that hinders acute care settings from reaching optimal levels of performance.^5^

Delayed discharges are very prominent worldwide. For example in the United Kingdom the marked increase in delayed discharges is of significant concern, especially after being linked with increased mortality rates.^6^ Likewise, a vast number of studies conducted throughout EU countries provided ample evidence of similar occurrences.^7–10^ This has led policy-makers and healthcare managers to address issues related to inpatients’ length of stay in an effort to cut down on costs and improve hospital patient flow and management.^11^

It tends to be very difficult to eradicate ‘delays’ because they are not always easily identifiable.^5^ The more one explores this issue in different country settings, the more it becomes apparent that the concept of a ‘delayed discharge’ lacks clarity and is in dire need of being properly defined. Unless a common definition of the term exists, there can be no credibility in comparisons between different research investigations conducted in different settings. Identifying the multiple manifestations of delayed discharges is key to the development of interventions and policies.

 A lack of a common definition of delayed discharges qualified as a major research gap in existing literature, a gap that this scoping review will strive to address. The review will analyse the derived literature and establish a baseline (conceptual and operational) for the term ‘delayed discharge.’ This will hopefully provide future research efforts with a point of reference and prevent incongruencies when it comes to using the term. Another research gap that the scoping review strives to address is the comparison of delayed discharge prevalence with hospital ward setting and the nature of health system funding. These issues have as yet not been addressed by existent research. The scoping review will also investigate causes and effects of delayed discharges, as well as interventions to counteract their impact in terms of reducing the number/rate of delays in a number of healthcare settings around the world.

## Methods

A scoping review of the literature was conducted to identify studies and investigations related to delayed discharges in acute hospital settings. A scoping review provides an overview of a broad topic, with research question/s on which the review is focused.^12^ We also decided to choose a scoping review because we were not seeking to answer one specific question, but rather to cover a broad area of research in an attempt to come up with an encompassing set of results (which is what such reviews are utilized for).^13^ Scoping reviews are also very efficient in determining the need for a systematic review on the subject.^14^

###  Sources

 The following electronic databases were searched: Cochrane, EBSCO, PubMed, PubMed Central, Medline, and Web of Science. A specifically designed government-provided link was utilized in a health department workstation, which gave us access to 4 databases at once (PubMed/PubMed Central, EBESCO, Medline, and Cochrane). Web of Science was used separately but with the same combination of keywords. The above-mentioned databases were chosen due to their strength and prominence in the health research arena. The keywords used were: ‘delayed discharges’ OR ‘delayed discharge’ AND ‘acute hospitals;’ ‘delayed discharges’ OR ‘delayed discharge’ AND ‘bed-blocking;’ ‘delayed discharges’ OR ‘delayed discharge’ AND ‘patient flow;’ ‘discharge delays’ AND ‘bed-blocking’/’patient flow;’ ‘alternate level of care’ AND ‘delayed discharge;’ ‘transition of care’ AND ‘delayed discharge.’ The term ‘patient flow’ was later replaced by ‘acute hospital patient flow,’ as the former was deemed to be too vague and generic. The search was conducted between January 2017 and March 2020 and covered literature ranging from 1990 to 2019.

 Each of the above keywords (or combination of keywords) were applied to the different databases specified above. Search lists were manually compared and contrasted. This procedure was very important as in this way studies which were not aligned with our review’s aims were eliminated from the search (by referring to the inclusion/exclusion criteria). Replicated search items were also discarded in this way. Certain studies with plenty of research relevance had to be set aside in the process so that our research criteria were adhered to. By weighing in on doubtful articles was pivotal in selecting/unselecting relevant research material.

###  Search Strategy

**Figure 1:**
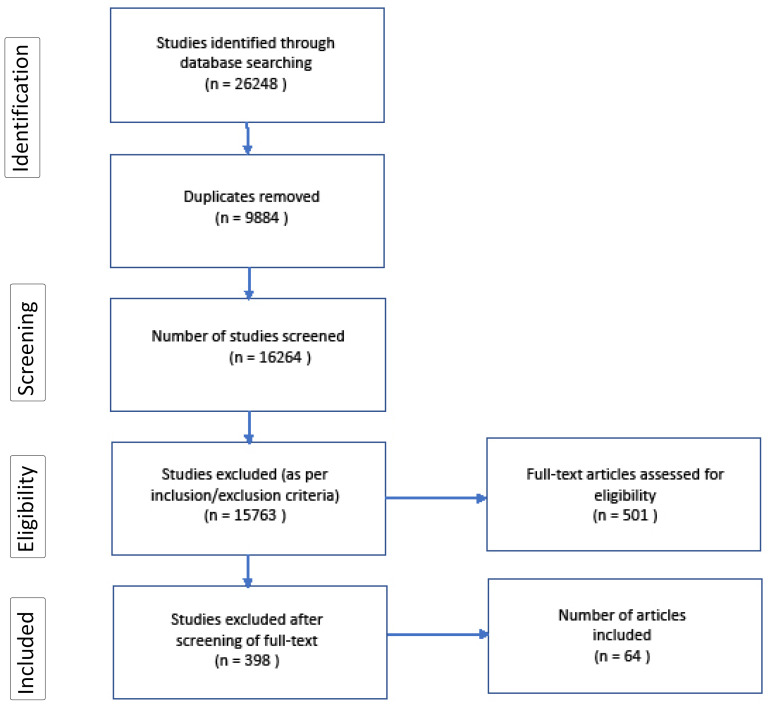
PRISMA Flow Diagram. Abbreviation: PRISMA, Preferred Reporting Items for Systematic Reviews and Meta-Analysis.

The PRISMA (Preferred Reporting Items for Systematic Reviews and Meta-Analysis) flow diagram below (Figure 1) provides a clear representation of the search results obtained, and how these findings were filtered to derive pertinent articles.^15^ An initial number of 26 248 articles were retrieved from the online database search. Duplicates were removed and inclusion/exclusion criteria (described below) were applied to derived abstracts.

 Articles were limited to those published in the English language between 1990 and 2019 (see Table). English was chosen as it is the working language of the reviewers. We limited our search to the period 1990-2019 in order to include the most relevant publications on delayed discharges of the last 30 years. The relevance of both article title and abstract was determined through authors’ collective agreement. Records were screened mainly for research setting (only acute hospital care facilities were included) and sample (adult population). This ensured that both setting and sample fit within the parameters of the research questions. In fact, some articles were excluded because studies were conducted in elderly homes, long-term residencies or psychiatric institutions. Studies which were carried out in paediatric or community-based settings were also excluded, ensuring that acute adult hospital settings remained the primary focus of the scoping review. This manual filtering of studies was effective in allowing us to extract pertinent investigations, and to prevent us from getting side-tracked into unrelated research areas. The systematic reviews’ reference lists uncovered in the search were also reviewed for new references/common references. Those articles which were deemed relevant (as they met the inclusion/exclusion criteria) to our scoping review were also included in the study. This helped greatly in preventing us from missing relevant information on delayed discharges.

**Table:** Inclusion/Exclusion Criteria

**Item**	**Inclusion Criteria**	**Exclusion Criteria**	**Justification**
Language	Articles written in the English language	Articles written in other languages	Scoping review authors/reviewers use English as their working language
Dating	Research articles published from 1990-2019	Articles published pre-1990 and post 2019	The delayed discharge phenomenon gained most prominence in the last three decades
Research setting	Only research conducted in acute hospital settings was included	Studies conducted in other areas were excluded	The research question focused primarily on acute hospital settings
Target population	Solely adult ward settings were included	Paediatric ward settings were excluded	Only adult settings will be tackled to prevent confusion and facilitate comparison of results
Evidence-base	Only primary evidence-based research articles were included	Opinion articles and other speculative write-ups were excluded	This was deemed to add rigor, strength and value to the scoping review (while avoiding bias)
Research perspective	Articles taken from an organisational perspective	Articles taken from patients’ perspective	Delayed discharge definition deemed to differ according to perspective. At present organisation (not patient) is the primary decider of care

###  Selection and Data Extraction

 The final set of articles (n = 64) selected for full review met specified research criteria. Reference lists of the chosen articles were also screened for other studies. These articles were divided into 3 subgroups, namely: Type A: systematic reviews/meta-analysis (n = 5); Type B: randomized controlled trials/experimental studies (n = 5); and Type C: descriptive studies/case studies (n = 54). This was done in an attempt to create a hierarchy of importance (evidence), with systematic reviews and experimental studies carrying the most weight. Information was derived from a wide variety of different journals, since there was not one specific journal type that focused on delayed discharges in particular. All pertinent data from the 64 selected articles were transferred to a separate data extraction form. This tool was developed in an effort to organise derived information into tabular form, paving the way for easy analysis and comparison. The data extracted from the abstracts/chosen studies included the aims of the studies, type of research methodology used, results obtained, and conclusion derived from the investigations. Data on sample size and research tools were also recorded. Attention was also given to the country the research was conducted in and the ward setting where it took place.

## Results

###  Building Conceptual and Operational Definitions for Delayed Discharges 

All articles included in this study were analysed in an effort to extract data related to conceptual and operational definitions of delayed discharges (see Supplementary file 1, Table S1). Other information was also extracted with the purpose of establishing a link between the definitions provided by different authors and country of origin, types of health systems, causes and effects of delayed discharges and healthcare costs (see Tables S1 and S2 – Supplementary file 1).

 From a conceptual viewpoint, a number of keywords – derived from a vast number of different (but similar) definitions – were singled out. This was done by comparing and contrasting all available construct definitions and by identifying the most common ones. These keywords were: extra hospital time (n = 1); inappropriate occupancy (n = 2); medically fit (n = 12); unable to leave (n = 6); timely hospital stay (n = 1); exceeding length of stay (n = 2); needless hospital admission (n = 2); lack/inadequate transfer of care arrangements (n = 6); health professionals’ convenience (n = 1); delayed examinations/investigations/treatment of patients (n = 5); and lack of information, miscommunication (n=2), transition of care (n = 2) and alternate level of care (n = 6). We therefore propose a comprehensive conceptual definition of inpatient delayed discharges as:

 An instance where a medically-fit patient is needlessly kept in hospital due to internal organisational/operational factors or where a patient is flagged as in need of alternate level of care and is delayed because of deferred transition of care and/or lack of external transfer-of-care arrangements.

 From an operational (measurement) viewpoint, defining delayed discharges was unique to some studies. These ranged from having patients leaving the hospital on the day of discharge at different time points, namely after 10:00 am, 11:00 am, midday, and up to 24 hours post-discharge. These definitions also included having patients leaving after 6:00 pm post day-care procedure. In one study, a delay was even defined to consist of anything exceeding 30 days post transfer of care. It is therefore extremely difficult to establish a standard operational definition of delayed discharges in view of the diverse viewpoints of how this construct is measured. However, it seemed to revolve around a more precise mathematically finite unit of measure. The absolute majority of studies used ‘days’ but there were some which utilized ‘hours’ or ‘days and hours.’ We also sought to uncover if there is any relationship between definitions of delayed discharges and the research setting/country where these studies were conducted. Most studies took place in the United Kingdom and the United States, but others were found from all around the globe (including Norway, Italy, Malta, Belgium, Singapore, Australia, Brazil, Sweden, and Portugal).

 Most studies were conducted either throughout one single hospital or involved a wide range of different hospitals in a specific geographical location. Only a handful tackled one specific ward setting. This made it extremely difficult to identify any links between definitions of delayed discharges and specific health settings. Some studies, in turn, did not provide a formal definition of the term at all. Such disparity, while allowing for a more thorough view of a wider spectrum of ward settings, prevented us from successfully establishing relationships between causes and effects of delayed discharges. Uncovering a relationship between launching initiatives to counteract delayed discharges (intervention studies) and the role of different health professionals involved was another issue that we addressed. Although discharge planning and timing were the main focus of such studies, the roles of health professionals in the process remain still undefined and unclear.

###  Causes and Effects of Delayed Discharges and Implemented Interventions 

**Figure 2:**
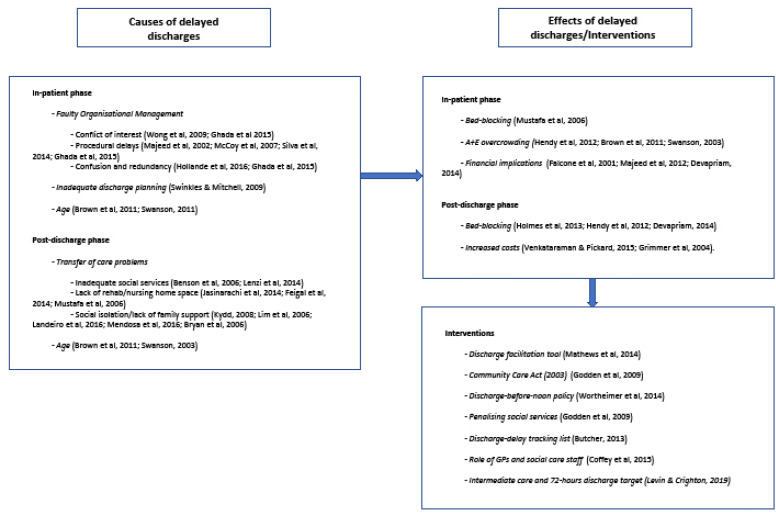
Systems Representation Derived From Scoping Review Findings.

The data extraction table was thoroughly analyzed in an effort to find common trends and differences, providing a set of results that adequately represent study findings. Categories were not pre-determined but were drawn up as the analysis progressed. A diagram (Figure 2), based on findings, provides a systems representation of delayed discharges of adult acute hospital patients.

Figure 2 outlines the main causes and effects of delayed discharges, which are classified into ‘in-patient’ and the ‘post-discharge’ phases. The diagram also presents the intervention studies addressing delayed discharges. These studies directly attempted to introduce operational mechanisms affecting the rate of delayed discharges in a particular acute hospital care setting. Some of these studies yielded positive, albeit modest, outcomes, and although derived results did not spur radical changes (percentage reduction in overall delays), they did seem to point towards specific courses of action (strategies aimed at reducing discharge time on the actual day of discharge). No apparent relationship between country/health system type and causes for delays in the discharge of patients from acute hospital settings emerged from results. Neither was there a link between ward setting and causes of delays. The causes described in the above diagram seemed to be present across various ward settings throughout the studies included in the scoping review.

## Discussion

###  Conceptual and Operational Definition of Delayed Discharges 

 The aims of this scoping review were to investigate the conceptual and operational definitions of delayed discharges, explore causes and effects of delayed discharges, and identify interventions aimed at decreasing the impact of delayed discharges in acute healthcare settings. Table S2 analyses the definitions utilised in the 64 articles included in this scoping review and distinguishes the conceptual from the operational aspect. It also provides details regarding the unit of measurement. Based on this scoping review, we propose the following conceptual definition of delayed discharges: An instance where a medically-fit patient is needlessly kept in hospital due to internal organisational/operational factors or where a patient is flagged as in need of alternate level of care and is delayed because of deferred transition of care and/or lack of external transfer-of-care arrangements.

 Operational definitions varied across the studies, mostly due to the different contexts of the study settings. It was therefore not possible to emerge with an encompassing common operational definition of delayed discharges. A marked difference in available published data may have also played a role in preventing the emergence of an overall operational definition, as opposed to conceptual cases where definitions exist within the boundaries of thought alone (even because the experiences of patients and health providers in relation to delayed discharges vary across the board).

 Nevertheless, we identified several commonalities between studies, even though these were conducted in different settings and in different countries. Operational definitions were mostly predominant to privately funded health systems (refer to Tables S1 and S2). This seems to suggest that for-profit health organisations are more focused on issues related to timely discharges and saving costs. This claim is supported by the finding that whereas studies yielding conceptual definitions (mostly conducted in publicly-funded health systems, such as the United Kingdom and Northern European settings) primarily made use of ‘days’ as the chosen unit of measure, operational studies (funded mostly by for-profit organisations) displayed a tendency for a higher level of precision (using days, hours, and even minutes in some cases as units of measure). This points towards a situation where profit-making triggers health organisations to apply a degree of mathematical precision in preventing delays with the aim of improving the discharge process, improving efficiency and potentially reducing waste in terms of bed occupancy. At this point in view of data availability variation, it would be of benefit to state that the relationships described above are not meant to provide any form of statistical significance as regards differences in accuracy of measurement between the two sectors. Our observation is merely based on the literature available in this review. In turn, more detailed data is observed in published papers investigating private providers due to easier access by researchers. Publicly available data of public hospitals is likely to be less detailed and therefore so are studies based on such data. This fact can be a major confounder when comparing public and private sector data.

The next sections will address the causes and effects of delayed discharges, together with interventions employed by specific health settings to counteract their impact. The diagram (Figure 2) summarizes the findings, as well as organizes and groups causes, effects, and interventions in relation to delayed discharges with the aim of testing relationships in future research between the various constructs identified.

###  Causes and Effects of Delayed Discharges

Research uncovered a tendency for the rate of delayed discharges to be particularly due to issues related to overall hospital organisation and management. The use of the Appropriateness Evaluation Protocol tool to assess for delays in the analysis of patients’ medical records in two separate hospitals concluded that lack of improvement in care team organisation was primarily responsible for the delays incurred.^16^ These findings were in line with studies conducted by other researchers.^17–21^ A number of problems were identified in this field, ranging from conflicts of interest between health professionals^11,22^ to procedural delays, mainly pertaining to waiting for tests and investigations,^19^ to mere confusion and redundancy in the plan of care.^19^ Such findings are a cause for concern, in that they seem to point towards health systems with inherent operational system failures. Issues such as ‘conflicts of interest’ and ‘procedural delays,’ unlike external independent variables like ageing and population epidemiology, are within hospital management’s grasp to control.

Delayed discharges were found to cause severe A&E (Accident & Emergency) overcrowding^23^ and bed-blocking,^5,24,25^ due to the increased inefficiency and waste that this phenomenon brings with it. This goes together with the inevitable negative financial implications typically incurred in such situations.^26–29^ In England, there were on average 4000 patients a day who experienced some form of delay in transfer of care between 2013 and 2016.^30^ This translated to approximately 115 000 bed days’ worth of delayed care. In turn, hospital patients who are medically fit for discharge cost the Scottish National Health Service (NHS) an extra £100 million over the course of 2016 (around £214 per patient per day). These figures concur with the ones incurred in England, where delays in the discharge of patients who are ready from treatment have topped £900 million per year.^30^

The lack of proper discharge planning was another factor that we identified as having a negative impact on an effective and timely discharge process,^31-34^ thereby directly resulting in both discharge delays as well as increased re-admission rates. This state of affairs is counter-productive, mainly because discharge planning should actually be used as a tool to curtail unnecessary delays. However, it is a factor that can be acted upon earlier on in the patient’s hospital stay (even as early as the admission phase).^35^ In fact, a specifically designed computer program to track delayed discharges in real-time in a large academic medical centre in the United States was utilized for this purpose.^35^ The study uncovered a tendency for inadequacies in the discharge process (ranging across the patient’s overall stay to the actual day of discharge) to be mainly responsible for incurred delays (mainly in the form of delayed paperwork and organisation related to transfer of care). These findings also seem to support the commonly shared perspective that acute hospital facilities attach limited importance to the discharge planning process, as they are more focused on the medical treatment provided rather than on what happens after treatment ends. Some authors also identified the discharge process, which takes place on the discharge date itself, as an occurrence resulting in delays.^36^

The post ‘medically discharge’ phase was mainly concerned with problems related to the transfer of care of patients who were deemed to be medically fit for discharge by the system. These problems involved: (*i*) lack of proper community services support,^9,37-39^ (*ii*) lack of adequate social services,^40,41^ (*iii*) no space in nursing homes and rehabilitation facilities,^8,42-45^ and (*iv*) social isolation due to lack of family support.^46,47^ This phase highlights the dependency of acute hospital settings on primary, as well as community, long-term and rehabilitative care. This is because, as far as the acute hospital facility is concerned, the patient has been duly cured and discharged, with all the processes involved therein meeting expectations. Unfortunately, with the ever present (albeit increasing) pressure exerted by ageing populations and associated high dependency levels, this phase of the discharge process is particularly prone to presenting challenges to hospital managers.

Two systematic reviews^48,49^ tackled delayed discharges from two perspectives: the first on older people, while the second on financial and logistical impact of delayed discharges on acute hospital care management. Both systematic reviews were spurred by rising concerns about the effects of an ageing population on the demand for acute hospital beds. Authors’ conclusions differed, in that while one claims there is weak evidence linking delayed discharges with the older persons,^49^ the other insists that age is indeed a determining factor.^48^ While the outcomes from these studies are indeterminate, ageing and associated morbidity as causes of delayed discharges seem to be gaining momentum. In addition, age (and related co-morbidities) – as a factor affecting the rate of delayed discharges by way of making discharge plans and transfer of care more challenging was also a conclusion reached by an analysis of 453 case notes over a 6-month period in an orthopaedic setting.^50^

In turn, a systematic review of 32 studies identified problems related to social services as being the main cause of delays.^51^ These mainly were insufficient care home capacity and community-based care. On the other hand, having good post-discharge planning and assessment (to prevent re-admissions),^52-54^ together with active engagement of general practitioners and other social care staff,^55,56^ was highlighted in this review as effective in the prevention of delays in discharge. These findings strengthen the need for developing a sound community-based framework for post-discharge care, specifically in the context of social support and dependency management in addition to medical-based community care. The impact of cost on acute hospital settings in the absence of such services is particularly pronounced^10,57^ due to the bed-blocking effects inevitably incurred.^5,28,58^

An additional two more recent systematic reviews pertaining to cause and effect of delayed discharges were analysed. One such review^59^ chose to tackle delayed discharges from a prevalence and cost perspective. This review uncovered a link between delayed discharges and morbidity and mortality in older people, especially due to iatrogenic infections. There was also found to be a link between high dependency and delayed discharges, with social isolation playing a major role. From a cost perspective the authors identified opportunity costs related to bed-blocking, waste, and A+E overcrowding to be the most prevalent. This systematic review concluded that the delayed discharge phenomenon was prevalent in most countries, with the average cost varying between $142 and $31 395. The study also identified the major causes of delay as being, (*a*) organisational factors, (*b*) lack of assessment and discharge planning, (*c*) poor communication between the organisation and the patient, and (*d*) insufficient statutory services. In turn, while providing financial incentives for the timely transfer of care worked well in Norway and Sweden, such methods failed in the United Kingdom. This last finding seems to be in line with the conclusions of another study,^31^ referring to the ineffectiveness of the Community Care Act of 2003 launched in the United Kingdom and aimed at penalising social services for delays in patient discharge from acute healthcare settings. Another systematic review^60^ tackled the issue of delayed discharges from an ‘impact and experience’ perspective. Findings uncovered a number of outcomes related to delayed discharges, namely (*a*) an impact on patient health outcomes (increased mortality, increased depression, increased dependency and associated decrease in activities of daily living), (*b*) an impact on staff (frustration and guilt, feeling that their patients were being dehumanized), and (*c*) an impact on the organisation (an increase in re-admission rates, a decrease in inter-professional communication, and added cost due to waste). The findings in the above-mentioned reviews (including our own scoping review) seem to be very congruent when it comes to cause and effect dynamics related to delays in patient discharge. This was an encouraging finding in our scoping review because it seems to point to a number of common denominators which health systems can address to counteract and ultimately overcome to lessen the occurrence of this phenomenon.

###  Interventions

A number of studies were conducted with the purpose of introducing specific measures (mainly ward/setting-based) to lessen the impact of delayed discharges in acute healthcare settings. One such study^36^ attempted to develop a discharge facilitation tool to aid in the promotion of early discharges, achieving an overall improvement of 10% in the rate of total discharges. Another intervention study by a group of researchers^41^ aimed to address this problem by attempting to introduce a ‘discharge before noon’ policy, which initiative turned out to be both possible and sustainable (with discharges before noon increasing from 11% to 38% over a 13-month period). In both cases there was a group effort from multiple members of the multi-disciplinary team, who were involved in documenting and tracking their progress through the day in a way as to allow us to identify potential instances that could lead to delays. Such techniques are fairly cheap to implement, requiring minimal staff and effort, but yield relatively positive outcomes.

One author^61^ even went one step ahead and created a tracking list with the intent of identifying factors that hinder early discharge (in a neurology setting). While this intervention study had no impact on patient length of stay, it resulted in an overall drop in 30-day re-admission rates and uncovered a mere 36.4% discharge rate occurring before 10:00 am. This study also attempted to create more re-enforcement and awareness among health professionals regarding a timelier discharge process on the actual day of discharge. From these studies it becomes evident that the involvement of health professionals is pivotal in the attainment of discharge delay prevention, mainly due to the fact that these individuals are the ones who are actually at the point of service and most likely to identify such instances. However, the majority of authors in this field agreed that problems related to delayed discharges are multi-dimensional and vary across the board.

The Community Care Act of 2003 introduced in England was designed to financially penalise social care facilities for delays in the transfer of care. This measure was deemed as being needed in view of increasing bed capacity insufficiency throughout acute hospital settings. A trend analysis of hospital activity between 2001 and 2007 was carried out,^62^ with the intent of assessing the impact of the Community Care Act of 2003 on delays in patient discharge. This however revealed that there was a lack of evidence that the Act somehow contributed to a decrease in delayed discharges, with the absolute majority of those same delays (68%) attributed to the NHS efficiency itself. Another study^63^ was conducted which aimed to measure the effect of intermediate care and a 72-hours discharge target on days delayed. Discharge delays were compared before and after the onset of intermediate care initiatives. Results yielded positive outcomes, in that there was an association between a reduction in delays and intermediate care combined with the 72-hour discharge target. Although such delays continued to increase over time, these increases were found to have been greater in the absence of this initiative.

###  Study Limitations and Recommendations for Research

 One study limitation revolves around the fact that we confined our scoping review to studies carried out within adult acute hospital care. We excluded geriatric settings, long-term facilities, paediatric settings as well as psychiatric hospitals. We thereby recommend any research that chooses to go beyond adult acute hospital care and explore other settings.

 Another limitation pertains to the relationship between various variables identified in the review data. Although in many cases we ventured to suggest possible cause and effect relationships, many of these are nonetheless not supported by the data extracted and thereby not conclusive. Further research is needed in the area to determine the veracity of such links (in terms of the statistical significance of their relationships).

 Our derived conceptual definition of delayed discharges is solely focused on adult acute hospital care from the organisation’s perspective. It does not include the patient’s perspective, for which we think a whole new conceptual definition would need to be drawn up. We decided that since most healthcare systems in developed and developing countries have not yet moved to a person-centred care approach (but rather still utilize a patient-centred care approach), a definition of delayed discharges from the organisation’s point of view would be of most benefit because at this point in time it is not the patient who ultimately determines the plan of care but the organisation itself (through the various health professionals). We highly recommend further research into the area in an attempt to uncover conceptual definitions of a delayed discharge from the patient’s perspective, which can form the basis for future healthcare models built around person-centred care.

## Conclusion

 This scoping review is intended to be a helpful precursor for future systematic reviews or other emerging approaches to evidence synthesis (such as realist reviews). It has, in turn, been used to confirm the relevance of the chosen inclusion criteria and potential questions on the subject of delayed discharges. It contributes to knowledge in that it provides a holistic definition that captures the full complexity of the construct and goes beyond what has been explicitly conceptually defined. In this scoping review, we have provided a comprehensive yet extensive picture of causes and effects in relation to delayed discharges of adult acute hospital patients. We proposed a conceptual definition of delayed discharges based on the derived literature, as well as a systems representation that distinguishes (yet links) causes and effects. In addition, we identified intervention studies that attempted to minimise the problem of delayed discharges. The comparing and contrasting of the different research investigations also yielded very valuable information that allowed us to identify very important relationships between specific variables. The relationships described above may not be statistically significant, meaning that more research in the area is warranted to establish causality. The fact that a clear-cut definition could not be drawn from an operational standpoint (due to such variance in the literature) is indicative of the complexity of the process involved and can be considered as a basis for further research in the area. The results of this scoping review as represented in the systems model may guide future research on delayed inpatient discharges. Aligning acute hospital settings with measures to prevent causes and implement changes to decrease the effect of delayed discharges is envisaged to minimise the problem, while aiming for a higher availability of hospital beds, less A&E overcrowding, strengthened partnerships between hospital and community care and, ultimately, a drop-in healthcare waste and related costs.

## Acknowledgements

 We acknowledge the valuable feedback of the editor and the four reviewers. The changes that we carried out as a result of the feedback have greatly improved the manuscript.

## Ethical issues

 Not applicable.

## Competing interests

 Authors declare that they have no competing interests.

## Authors’ contributions

 Conceptualization: AM, SB; Data curation: AM; Formal analysis: AM; Supervision: SB, LG, GT; Roles/Writing – original draft: AM, SB; Writing – review and editing: SB, GT.

## Authors’ affiliations

^1^Department of Health Services Management, Faculty of Health Sciences, University of Malta, Msida, Malta. ^2^Department of Computer Information Systems, Faculty of ICT, University of Malta, Msida, Malta.

## Supplementary files


Supplementary file 1 contains Tables S1-S2.
