# Supplementary material for: Defining Delayed Discharges of Inpatients and Their Impact in Acute Hospital Care: A Scoping Review
Source: Int J Health Policy Manag. 2020 Jun 29;11(2):103–11. doi: 10.34172/ijhpm.2020.94 (PMC9278600; doi:10.34172/ijhpm.2020.94)
Supplement: Supplementary file 1 — contains Tables S1-S2. [file ijhpm-11-103-s001.pdf]

Supplementary file 1

Table S1. The Conceptual and Operational Definitions of Delayed Discharges With Units of Measurement

Author/date	Type of study	Conceptual	Operational	Definition of delayed discharges	Units of measure
Adams et al (2015)	Semi-structured interviews		Operational	A delayed discharge ensued when a number of patient and provider factors resulted in more timely hospital stays and increased bed use	Not specified
Bai et al, 2019	Record Analysis	Conceptual		No formal definition provided	N/A
Baumann et al (2007)	Record analysis	Conceptual		No formal definition provided	Not specified
Benson et al (2006)	1-day survey data collection	Conceptual		Patients who are unable to be discharge from ward despite being fit to leave	Days
Brown et al (2011)	Patient record analysis	Conceptual		Patients who were unable to be discharged despite being fit to leave hospital	Days
Bryan et al (2006)	Record analysis / manager interviews	Conceptual		Delays in moving the patient through the care chain after medical discharge from hospital	Not specified

Author/date	Type of study	Conceptual	Operational	Definition of delayed discharges	Units of measure
Bryson (2011)	Survey study	Conceptual		Patients who had been declared medically fit for discharge	Not specified
Butcher (2013)	Intervention study		Operational	A discharge that happened after 10am on the day the patient left the hospital	Hours
Challis et al (2014)	Record analysis	Conceptual		A situation where a patient is deemed to be medically well enough for discharge but they are unable to leave hospital because arrangements for continuing care have not been finalized.	N/A
Coffey et al (2015)	Systematic Review	Conceptual		A situation where a patient is deemed to be medically well enough for discharge but is unable to leave hospital because arrangements for continuing care have not been finalized	N/A
Costa et al (2012)	Record analysis	Conceptual		A hospital episode where a patient exceeds the length of stay deemed necessary	Days
Devapriam et al (2014)	Record analysis	Conceptual		A situation where a patient is deemed to be	Days

Author/date	Type of study	Conceptual	Operational	Definition of delayed discharges	Units of measure
				medically well enough for discharge but they are unable to leave hospital because arrangements for continuing care have not been finalized.	
Edirimanne et al (2010)	Survey audit		Operational	The difference between expected date and time of discharge and actual date and time of discharge	Days/hours
Falcone et al (1991)	Record analysis	Conceptual		A period between the day the patient was judged to be medically discharged and the day he/she actually left the hospital	Days
Feigal et al (2014)	Survey audit	Conceptual		Hospitalizations that no longer meet acute in-patient care or care needs	Days
Fontaine et al (2011)	3 surveys over 3 years	Conceptual		Defined as per list of criteria expressed on the Appropriateness Evaluation Protocol (AEP)	Days
Ghada et al (2015)	Before and after study		Operational	Target discharge time set at 105 minutes (20% reduction from	Days/hours/minutes

Author/date	Type of study	Conceptual	Operational	Definition of delayed discharges	Units of measure
				baseline historical data)	
Glasby et al (2004)	Literature Review	N/A	N/A	No formal definition provided (authors draw attention to lack of clarity in defining the term)	N/A
Godden et al (2009)	Record analysis	Conceptual		As per Community Care Act 2003: a delay for reasons related to community care services	Days
Goughan et al (2015)	Record analysis	Conceptual		When a patient is medically ready to be discharged and cared for in another setting but is unable to do so	Days
Green et al, 2017	Record Analysis	Conceptual		When an admitted patient would be discharged from the care they were receiving but was still occupying a bed	N/A
Hendy et al (2012)	Real-time record analysis		Operational	The sum of delays that prolonged a patient's hospital stay	Days/hours
Holland et al (2016)	Real-time electronic delay tacking	Conceptual		A delay occurs when a patient's discharge occurs after the time-	Days

Author/date	Type of study	Conceptual	Operational	Definition of delayed discharges	Units of measure
				point established between the provider and the patient	
Holmes et al (2013)	Record analysis	Conceptual		Patients who no longer require acute care, who are occupying acute care beds while awaiting lower-level placement	Days
Jasinarachi et al (2009)	Observational study		Operational	When a delay lasts more than 24 hours after the patient is deemed to be medical fit	Days
Kydd (2008)	Participant observation/interviews	Conceptual		When a patient is inappropriately occupying a hospital bed	Not specified
Laugaland et al (2014)	Participant Observation		Operational	No formal definition is available.	Hours/Days
Lim et al (2006)	Record analysis		Operational	Patients with a length of hospital stay of 28 days or more (in a context of an average length of stay of 10.9 days)	Days
Landeiro et al (2016)	Prospective study of admitted ward patients	Conceptual		Old people who were deemed medically fit for discharge post-hip fracture treatment but were unable to leave hospital	Days

Author/date	Type of study	Conceptual	Operational	Definition of delayed discharges	Units of measure
				due to social isolation	
Landeiro et al, 2019	Systematic Review	Conceptual		Occurs when a hospital inpatient has been deemed medically fit for discharge but continues to occupy a hospital bed for non-medical reasons	N/A
Lenzi et al (2014)	Record analysis	Conceptual		When medically fit patients are unable to leave hospital due to unfinalized continuity of care arrangements	Days
Levin & Crighton, 2019	Time series analysis		Operational	No formal definition provided	Hours/days
Majeed et al (2012)	Record analysis	Conceptual		Inappropriate occupancy of hospital beds (divided into pre/post discharge)	Days
Mathews et al (2014)	Intervention study		Operational	A discharge which occurs after 11am on the day of discharge	Days/hours
McCoy et al (2007)	Record analysis	N/A	N/A	No formal definition specified	Days
Mendosa et al (2012)	Observational study	Conceptual		When a patient is considered medically fit for discharge but	Days

Author/date	Type of study	Conceptual	Operational	Definition of delayed discharges	Units of measure
				continues occupying a bed due to non-medical problems	
Mustafa et al (2016)	Record analysis	Conceptual		A patient who remains in hospital after a senior doctor (consultant or registrar grade) has documented in the medical chart that the patient can be discharged	Days
Nardi et al (2007)	Record analysis	Conceptual		Situations involving an economic, human and organizational burden exceeding patient's and their family's capacities, inducing a delay	Days
Nicholas et al (2002)	Record analysis	Conceptual		Defined as the number of day from when the patient is no longer in need of acute medical in-patient care to eventual discharge	Days
Philp et al (2013)	Systematic Review	N/A	N/A	No formal definition provided	N/A
Rambani & Okafor (2008)	Record analysis	Conceptual		The extra hospital time spent by patients after discharge	Days

Author/date	Type of study	Conceptual	Operational	Definition of delayed discharges	Units of measure
				post-acute orthopedic trauma	
Rajas-Garcia et al, 2017	Systematic Review	Conceptual		A period of continued hospital stay after a patient is deemed fit to leave hospital but is unable to do so for non-medical reasons	N/A
Rohatgi et al, 2018	Record analysis	Conceptual		No formal definition provided	N/A
Sant et al (2015)	Observational Study		Operational	When a discharge occurred after 6pm on the same day as the day-care procedure	Hours/Days
Silva et al (2014)	Record analysis	Conceptual		Defined as per item list in Appropriateness Evaluation Protocol (AEP)	Days
Solange-Reyes et al, 2017	Record Analysis	Conceptual		No formal definition provided	N/A
Styrborn & Thorslund (1993)	Record analysis	Conceptual		A patient judged by the physician responsible as being medically ready for discharge but who cannot leave hospital because of alternative forms of care or	Number of bed-blocking patients

Author/date	Type of study	Conceptual	Operational	Definition of delayed discharges	Units of measure
				because of social circumstances	
Swanson (2013)	Before and after study	Conceptual		When a patient no longer requires acute hospital care but remains in the hospital due to a variety of reasons	Days
Swinkles & Mitchell (2009)	Conversational interviews	N/A	N/A	No formal definition available	N/A
Venkataraman & Pickard (2015)	Record analysis		Operational	Delays affected due to waiting for INR to stabilize as a result of warfarin therapy	Days
Victor et al (2000)	Record analysis	Conceptual		A patient who is not discharged on the day that the consultant decides that he/she is medically fit for discharge	Days
Wong et al (2009)	Record analysis	Conceptual		No formal definition provided	Days
Williams et al (2010)	Before and after study		Operational	Discharge was considered delayed if the patient was not relocated from the ICU within 8 hours of being considered eligible for discharge by ICU medical staff	Days/hours

Author/date	Type of study	Conceptual	Operational	Definition of delayed discharges	Units of measure
Wortheimer et al (2014)	Intervention study		Operational	A discharge that happened after midday on the day the patient left the hospital	Hours
Worthington & Oldham (2006)	Survey study		Operational	Defined as a delay of more than 30 days before transfer of care	Days

Table S2. Analysis of Articles in Scoping Review by Country, Health Setting, Definition, Main Cause and Healthcare Cost

Country	Author	Health setting	Definition of delayed discharges	Main cause for delayed discharge	Public/Private funding (%) / Cost of health care per capita/year
**Canada**	Adams et al (2015)	Cardiac department in Sunnybrook Health Sciences Centre	A delayed discharge ensued when a number of patient and provider factors resulted in more timely hospital stays and increased bed use	Inability to construct proper follow-ups and home care services	Public funding (71%)<br>Private funding (29%)<br>Cost per capita/year: €3,749
	Costa et al (2012)	A large Canadian health region	A hospital episode where a patient exceeds the length of stay deemed medically necessary	Multiple co-morbidities and abusive behaviors	
	Wong et al (2009)	General Medicine	No formal definition provided	Health professionals' convenience caused delays	
	Bai et al (2019)	A wide range of hospitals	N/A	N/A	
**United Kingdom**	Benson et al (2006)	General surgery	Patients who are unable to be discharged from ward despite being fit to leave	Lack of intermediate care and social service provision	Public funding (86%)<br>Private funding (14%)<br><br>Cost per capita/year: €2,787
	Bryan et al (2006)	A hospital wide study	Delays in moving the patient through the care chain after medical discharge from hospital	Financial and organizational problems/Family resistance	
	Godden et al (2009)	A wide range of hospitals	As per Community Care Act 2003: a delay for reasons related to community care services	Bed-shortage and high re-admission rates	
	Goughan et al (2015)	A wide range of hospitals	When a patient is medically ready to be discharged and cared for in another setting but is unable to do so	N/A	

Country	Author	Health setting	Definition of delayed discharges	Main cause for delayed discharge	Public/Private funding (%) / Cost of health care per capita/year
	Green et al (2017)	A hospital wide study	Where an admitted patient would be discharged from the care they were receiving but were still occupying a bed	Lack of social services, delays in organizational processes	
	Hendy et al (2012)	Gastro-enterology medical team patients	The sum of delays that prolonged a patient's hospital stay	Patient's age	
	Levin & Crighton (2017)	A hospital wide study	No formal definition provided	Social factors, lack of discharge planning	
	Devapriam et al (2014)	Intellectual disability unit	A situation where a patient is deemed to be medically well enough for discharge but they are unable to leave hospital because arrangements for continuing care have not been finalized.	Delays in the completion of future needs assessment	
	Jasinarachi et al (2014)	Geriatric ward setting	When a delay lasts for more than 24 hours after the patient is deemed medically fit	Waiting for a nursing home/ waiting for proper assessment	
**United Kingdom**	Majeed et al (2012)	General surgery setting	Inappropriate occupancy of hospital beds	Delayed investigations/inadequate discharge arrangements	
	Rohatgi et al (2018)	A hospital wide study	N/A	Health professional delays, delays due to weekends	
	McCoy et al (2007)	A wide range of hospitals	No formal definition specified	Delays in treatment provision and lack of social support	
	Nicholas et al (2002)	A wide range of hospitals	Defined as the number of days from when the patient is no longer in need of acute medical in-patient care to eventual discharge	Patient dependency levels / finding suitable accommodation post-discharge	

Country	Author	Health setting	Definition of delayed discharges	Main cause for delayed discharge	Public/Private funding (%) / Cost of health care per capita/year
	Rambani & Okafor (2008)	A hospital wide study	The extra hospital time spent by patients after discharge post-acute orthopedic trauma	Age and related co-morbidities / social issues	
	Swinkles & Mitchell (2009)	A wide range of hospitals	No formal definition specified	Lack of information given to patients / co-morbidities / discharge intricacies and complicated planning process	
	Venkataraman & Picard (2015)	General surgery	Delays affected dur to waiting for INR to stabilize as a result of warfarin therapy	Lack of community based care of warfarin-related complications	
	Victor et al (2000)	A wide range of hospitals	A patient who is not discharged on the day that the consultant decides that he/she is medically fit for discharge	Absence of family carer, unavailable nursing home bed, lack of staffing for discharge assessment team	
	Worthington & Oldham (2006)	A wide range of hospitals	Defined as a delay of more than 30 days before transfer of care	No suitable placement / no funding for post-discharge support / poor mobility	
	Baumann (2007)	A wide range of hospitals	No formal definition provided	Shortage of staff/services	
	Kydd (2008)	Geriatric ward	When a patient is inappropriately occupying a hospital bed	Lack of nursing home space / lack of family support	
**Australia**	Brown et al (2011)	A wide range of hospitals	Patients who are unable to be discharged despite being fit to leave	Age was found to be a strong determinant in preventing timely discharge	Public funding (69%)<br>Private funding (31%)<br>Cost per capita/year: €3,331
	Edirimanne et al (2010)	Vascular surgery	The difference between expected data and time of discharge and actual date and time of discharge	Organisational factors, rehab facility inadequacy, and the age factor	

Country	Author	Health setting	Definition of delayed discharges	Main cause for delayed discharge	Public/Private funding (%) / Cost of health care per capita/year
	Solange-Reyes (2017)	A hospital wide study	N/A	Lack of residential care, administrative dekays and lack of social support	
	Williams et al (2010)	Intensive care unit	Discharge was considered delayed if the patient was not relocated from the ICU within 8 hours of being considered eligible for discharge by ICU medical staff	Lack of beds / delay in bed availability in transferring wards	
**United States**	Butcher (2013)	Neurology department	A discharge that happened after 10am on the day the patient left the hospital	Health professional (mainly physician) performance	Public funding (45%)<br>Private funding (55%)<br>Cost per capita/year: €7,508
	Falcone et al (1991)	A wide range of hospitals	A period between the day the patient was judged to be medically discharged and the day he/she actually left the hospital	Patients' age and social background/support	
	Feigal et al (2014)	A hospital wide study	Hospitalisations that no longer meet acute in-patient criteria or care needs	No home was available for patients incurring delays	
	Holland et al (2016)	A hospital wide study	A delay occurs when a patient's discharge occurs after the time-point established between the provider and the patient	Miscommunication among professionals, delay is discharge summary completion and discharge medication papers	
	Mathews et al (2014)	General medicine department	A discharge which happens after 11am on the day of discharge	N/A	
	Wortheimer et al (2014)	Neurology setting	A discharge that happened after midday on the day the patient left the hospital	N/A	

Country	Author	Health setting	Definition of delayed discharges	Main cause for delayed discharge	Public/Private funding (%) / Cost of health care per capita/year
**Belgium**	Fontaine et al (2011)	A wide range of hospitals	Defined as per list of criteria on the Appropriateness Evaluation Protocol (AEP)	Waiting for examination and lack of community-based services	Public funding (79%) Private funding (21%) Cost per capita/year: €3,667
**Italy**	Lenzi et al (2014)	A wide range of hospitals	When medically fir patients are unable to leave hospital due to unfinalized continuity of care arrangements	Waiting for bed in rehab/ lack of home support/ Age / Multiple co-morbidities	Public funding (81%) Private funding (19%)
	Nardi et al (2007)	A hospital wide study	Situations involving an economic, human and organizational burden exceeding patients' and their family's capacities, inducing a delay	Patient's age and dependency levels/degree of social support	Cost per capita/year: €2,651
**Norway**	Laugaland et al (2014)	Geriatric, medical and orthopedic ward settings	No formal definition is available	Timing, duration, and precision issues	Public funding (85%) Private funding (15%) Cost per capita/year: €5,050
	Swanson (2013)	A wide range of hospitals	When a patient no longer requires acute hospital care but remains in the hospital due to a variety of reasons	Patient's age	
	Holmes et al (2013)	A wide range of hospitals	Patients who no longer require acute care, who are occupying acute care beds while awaiting lower-level placement	Lack of social care services	
**Malta**	Sant et al (2015)	Day-care surgery unit	When discharge occurred after 6pm on the same day as the day-care procedure	Procedures taking longer than planned / Post-op complications / Organizational setbacks	Public funding (69%) Private funding (31%) Cost per capita/year: €2,647

Country	Author	Health setting	Definition of delayed discharges	Main cause for delayed discharge	Public/Private funding (%) / Cost of health care per capita/year
**Brazil**	Silva et al (2014)	A hospital wide study	Defined as per item list in the Appropriateness Evaluation Protocol (AEP)	Awaiting test results / awaiting professional consultations	Public funding (57%)<br>Private funding (43%)<br>Cost per capita/year: €1,267
**Singapore**	Lim et al (2006)	Geriatric medicine	Patients with a length of stay of 28 days or more	Social issues / lack of community support / nosocomial infections	Public funding (31%)<br>Private funding (69%)<br>Cost per capita/years: €2,372
**Portugal**	Landeiro et al (2016)	Rehabilitation unit	Old people who were deemed medically fit for discharge post-hip fracture treatment but were unable to leave hospital due to social isolation	Social isolation and lack of public funding	Public funding (67%)<br>Private funding (33%)<br>Cost per capita/year: €2,166
